# Linked‐Acceptor Type Conjugated Polymer for High Performance Organic Photovoltaics with an Open‐Circuit Voltage Exceeding 1 V

**DOI:** 10.1002/advs.201500021

**Published:** 2015-03-13

**Authors:** Benzheng Xia, Kun Lu, Yifan Zhao, Jianqi Zhang, Liu Yuan, Lingyun Zhu, Yuanping Yi, Zhixiang Wei

**Affiliations:** ^1^ National Center for Nanoscience and TechnologyBeijing100190P. R. China; ^2^ Institute of ChemistryChinese Academy of SciencesBeijing100190P. R. China

**Keywords:** high open‐circuit voltage, linked‐acceptor, polymer solar cells

## Abstract

A linked‐acceptor type conjugated polymer is designed and sythesized based on 4,8‐bis(5‐(2‐ethylhexyl)thiophen‐2‐yl)benzo[1,2‐*b*:4,5‐*b′*]dithiophene (BDTT) and linked‐thieno[3,4‐*c*]pyrrole‐4,6‐dione (LTPD). This polymer uses alkyl‐substituted thiophene as a bridge. The PBDTT‐LTPD includes two TPD units in one repeating unit, which can enhance acceptor density in the polymer backbone and lower the highest occupied molecular orbital (HOMO) level. By contrast, variable alkyl substitutions in the thiophene‐bridges ensure the subtle regulation of polymer properties. The solar cells based on PBDTT‐LTPD display an open‐circuit voltage (*V*_oc_) that exceeds 1 V, and a maximum power conversion efficiency (PCE) of 7.59% is obtained. This PCE value is the highest for conventional single‐junction bulk heterojunction solar cells with *V*_oc_ values of up to 1 V. Given that PBDTT‐LTPD exhibits a low HOMO energy level and a band gap equivalent to that of poly(3‐hexylthiophene), PBDTT‐LTPD/phenyl‐C_61_‐butyric acid methyl ester may be a promising candidate for the front cell in tandem polymer solar cells.

## 1 Introduction

Polymer solar cells (PSCs) that are flexible, lightweight, and low‐cost have attracted much attention because of their potential application as sustainable energy sources.^1^, ^2^, ^3^, ^4^, ^5^ To attain high power conversion efficiency (PCE) in such cells, efforts focus on synthesizing new materials, optimizing processing techniques, and utilizing new device architectures. Bulk heterojunction (BHJ) is the most efficient cell structure. It contains polymers or small molecule semiconductors as donors and soluble fullerene derivatives as acceptors. Donor materials in the backbone, which consist of alternating electron‐rich donor (D) and electron‐deficient acceptor (A) groups, facilitate the adjustment of the material properties.^6^, ^7^, ^8^, ^9^, ^10^ Following a series of evolutions, the PCE of PSCs based on D–A‐type materials has increased to over 9% in the past two years.^11^, ^12^, ^13^, ^14^

The alteration of different D or A groups is the general strategy used to obtain high‐performance molecules. Such molecules can be generated by extending the fused rings of D or A groups.^15^, ^16^, ^17^, ^18^, ^19^ However, the process of synthesizing both the extended D and A groups is typically very difficult. Moreover, the product yield is low. Some groups have also employed random polymerization to use three or more different monomers, which generally induces uncertainty in material energy levels and complicates property control.^20^, ^21^, ^22^ By contrast, some research groups have also attempted to synthesize conjugated polymers that contain two donors or two acceptors in one repeating unit. Adding up the acceptor units can effectively and simultaneously reduce the lowest unoccupied molecular orbital (LUMO) and the highest occupied molecular orbital (HOMO) levels by increasing acceptor density in the polymer backbone.^23^, ^24^ A low HOMO level significantly increases the open‐circuit voltage (*V*_oc_) of the PSCs. The strategy of linking two acceptors in one monomer and incorporating them into the polymer backbone was recently recognized for its effective tuning of energy levels, of charge transport properties, and of the absorption spectra of photovoltaic materials.^25^, ^26^, ^27^, ^28^

**Scheme 1:**
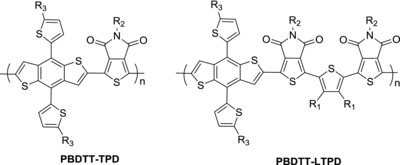
Molecular structures of polymers with single and linked acceptors. R_1_ = hexyl, R_2_ = octyl, R_3_ = 2‐ethylhexyl.

Thieno[3,4‐*c*]pyrrole‐4,6‐dione (TPD) has been widely applied in donor materials because of its strong electron‐withdrawing effect. Thus far, TPD has acted as a good acceptor for polymers, and the PCEs of their devices have exceeded 8%.^29^, ^30^, ^31^, ^32^, ^33^, ^34^ In our previous work, 4,8‐Bis(5‐(2‐ethylhexyl)thiophen‐2‐yl)benzo[1,2‐*b*:4,5‐*b*′]dithiophene (BDTT) and TPD were combined (**Scheme** **1**). This combination displayed moderate photovoltaic properties, with *V*_oc_ values of approximately 0.99 V. However, the current density (*J*_sc_) of the device was only 8.70 mA cm^−2^.^35^ Therefore, a hexyl‐thiophene unit was consequently introduced into this BDTT and TPD combination as a conjugated bridge to increase the absorption coefficient of polymers. The result demonstrated that this method can efficiently improve *J*_sc_ value to 10.94 mA cm^−2^ but that it reduces the *V*_oc_ slightly from 0.99 to 0.92 V.^36^ The addition of the electron‐rich thiophene unit increases donor density, which in turn lowers *V*_oc_ value. By contrast, Beaujuge and co‐workers reported that several high‐*V*_oc_ polymers show low *J*_sc_ and low PCE in BHJ PSCs.^37^ Additional research indicates that the inefficient transfer of holes from the fullerene to the polymer donor lowers internal quantum efficiencies and significantly reduces the device photo­currents and fill factors (FFs).^38^, ^39^, ^40^

In the current work, a linked‐acceptor is introduced into the polymer backbone to increase acceptor density and the absorption coefficient simultaneously. BDTT and linked‐thieno[3,4‐*c*]­pyrrole‐4,6‐dione (LTPD) are combined with hexylthiophene as a bridge. A novel PBDTT‐LTPD polymer is thus obtained (Scheme 1). The introduction of the LTPD unit is expected to enhance the electron‐withdrawing capability of the monomer. The dense alkyl‐side chain in the thiophene bridge ensures good solubility and aggravates the twisting of the polymer backbone. This twisting may also lower the HOMO level more than single acceptor polymers can.^41^, ^42^, ^43^ Thus, the theoretical calculation and experimental results demonstrate that the linked acceptors in the backbone can reduce HOMO level and increase the absorption coefficient considerably. The PSCs fabricated with this polymer exhibited a high *V*_oc_ value of 1.02 V with [6,6]‐phenyl‐C_61_‐butyric acid methyl ester (PC_60_BM). This *V*_oc_ value is among the highest reported thus far.^44^, ^45^ The obtained PCE reaches 7.59% with [6,6]‐phenyl‐C_71_‐butyric acid methyl ester (PC_70_BM). Moreover, *J*_sc_ value is 14.32 mA cm^−2^, which is a significant improvement over that of the single acceptor polymer‐based devices we reported. As far as we know, this PCE value is the highest for conventional single‐junction BHJ solar cells with *V*_oc_ values of up to 1 V.

## 2 Synthesis and Characterization

**Scheme 2:**
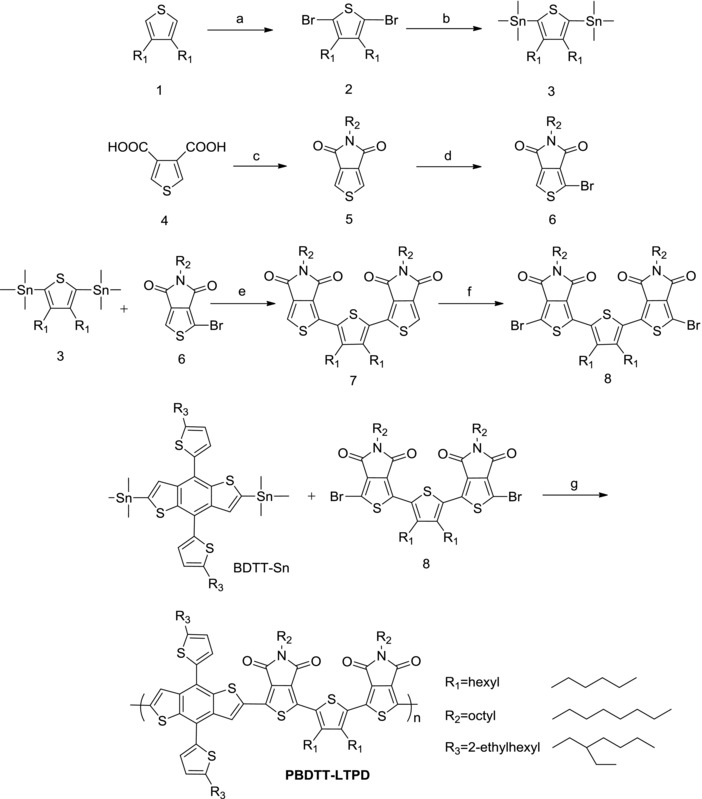
Synthetic route for the linked‐acceptor type monomers and PBDTT‐LTPD. a) CH_3_COOH:CHCl_3_ (1:10, v:v), NBS, room temperature, 3 h; b) *n*‐butyllithium, THF, −78 °C, 3 h, then chlorotrimethylstannane, −78 °C for 1 h, room temperature over night; c) acetic anhydride, 140 °C, overnight, toluene, *n*‐octylamine refluxed for 24 h, then thionyl chloride refluxed for 4 h; d) sulfuric acid: trifluoroacetic acid (1:10, v:v), NBS, room temperature, 10 min; e) toluene, Pd(PPh_3_)_4_, 95 °C under argon atmosphere, overnight; f) trifluoroacetic acid, NBS, ambient temperature, 4 h; g) toluene (10 mL) and DMF (2 mL), Pd (PPh_3_)_4_, 100 °C, 10 h under argon atmosphere.

Polymer PBDTT‐LTPD was synthesized through Stille‐coupling reactions (**Scheme** **2**). The molecular structures of all intermediates were characterized by proton nuclear magnetic resonance (^1^H NMR) and matrix‐assisted laser desorption‐ionization time‐of‐flight mass spectrometry (MALDI‐TOF‐MS) spectra. The donor and acceptor monomers were characterized further by ^13^C NMR spectra. The polymer was purified through column chromatography with chloroform as the eluent to obtain the product in the form of a dark solid. The results of high‐temperature gel permeation chromatography measurement with 1,2,4‐trichlorobenzene as the eluent (140 °C) showed a number‐average molecular weight (*M_n_*) of 15.6 kDa, with a polydispersity index (PDI) of 2.32 (Supporting Information, Figure S1). Theoretical calculation, UV–vis absorption, cyclic voltammetry (CV), differential scanning calorimetry (DSC, Supporting Information, Figure S4), space‐charge‐limited current (SCLC) measurements, atomic force microscopy (AFM), and grazing incidence wide‐angle X‐ray scattering (GIWAXS) were conducted to verify the properties of this type of linked‐acceptor polymer.

## 3 Result and Discussion

**Figure 1:**
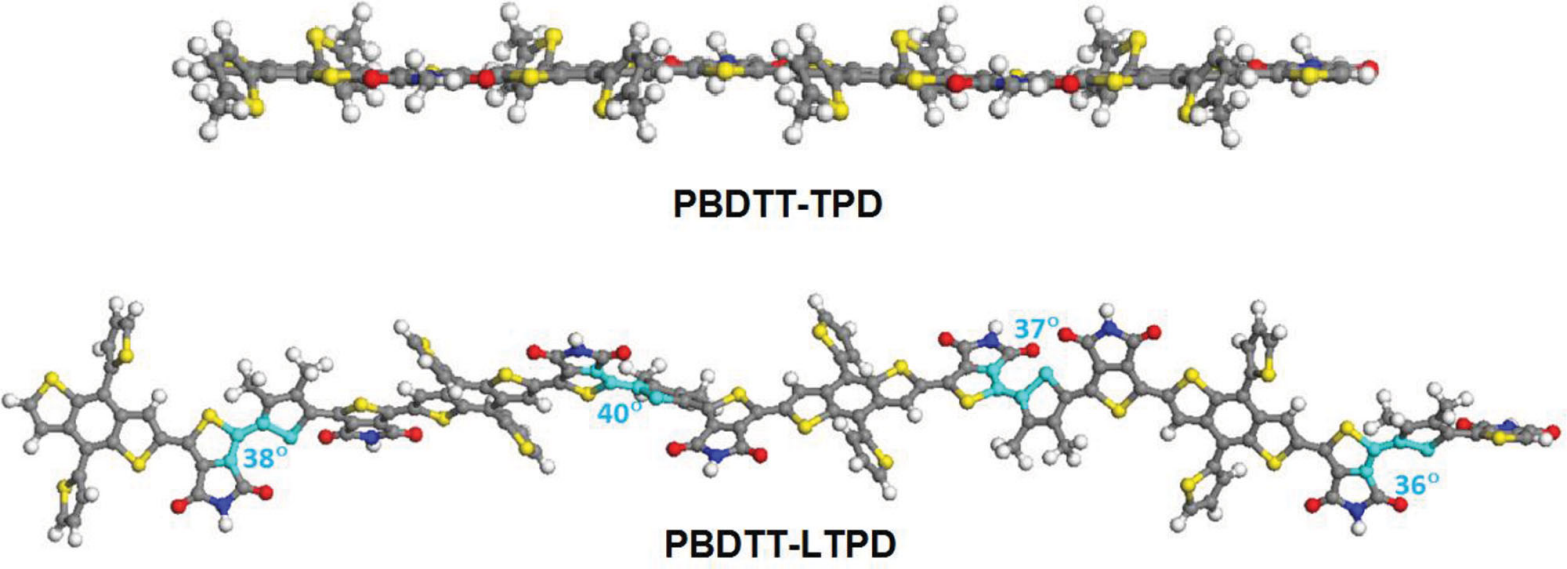
B3LYP/6–31G (d, p)‐optimized ground‐state geometries of the oligomers of PBDTT‐TPD and PBDTT‐LTPD tetramers.

**Figure 2:**
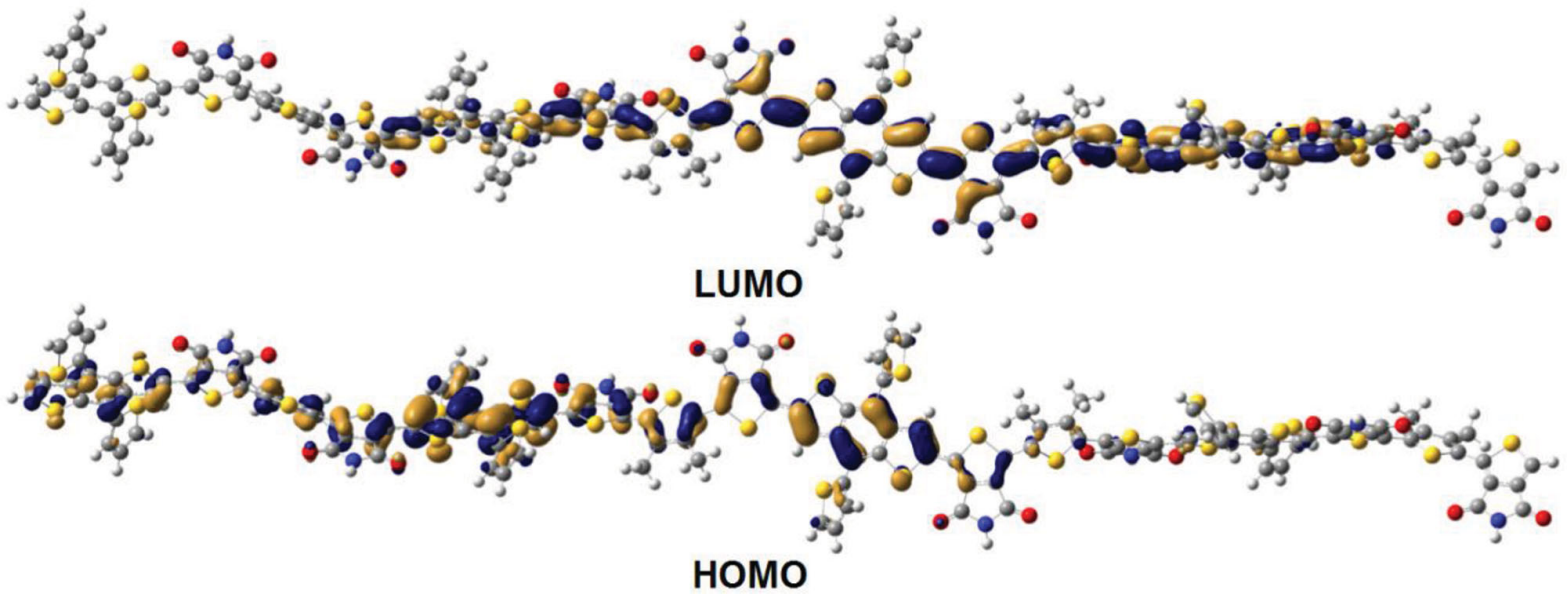
Frontier molecular orbital for PBDTT‐LTPD tetramers evaluated at the B3LYP/6–31G (d, p) level.

To gain insight into the fundamentals of the subtle change in molecular architecture from a single acceptor to a linked‐acceptor, the differences between PBDTT‐TPD and PBDTT‐LTPD were calculated theoretically and compared. During the calculations, the alkyl groups were replaced by hydrogen and methyl groups given that the backbone geometries of short oligomers do not vary substantially. The optimized geometries (**Figure** **1**) indicated that the electron‐donating BDT and the withdrawing TPD moieties on the backbone were almost fully coplanar in PBDTT‐TPD. In the case of PBDTT‐LTPD, the BDT and TPD moieties on the backbone remained almost planar. However, the thiophene and TPD moieties in the backbone were twisted at approximately 40°. As shown in **Figure** **2**, the HOMO and LUMO wave functions of the PBDTT‐LTPD tetramer were delocalized on both donor and acceptor units. Correspondingly, the calculated HOMO energy of the PBDTT‐LTPD oligomer was reduced by 0.2 eV in comparison with that of PBDTT‐TPD. This finding suggests that the *V*_oc_ value of the PBDTT‐LTPD oligomer is most likely greater than that of PBDTT‐TPD.

**Figure 3:**
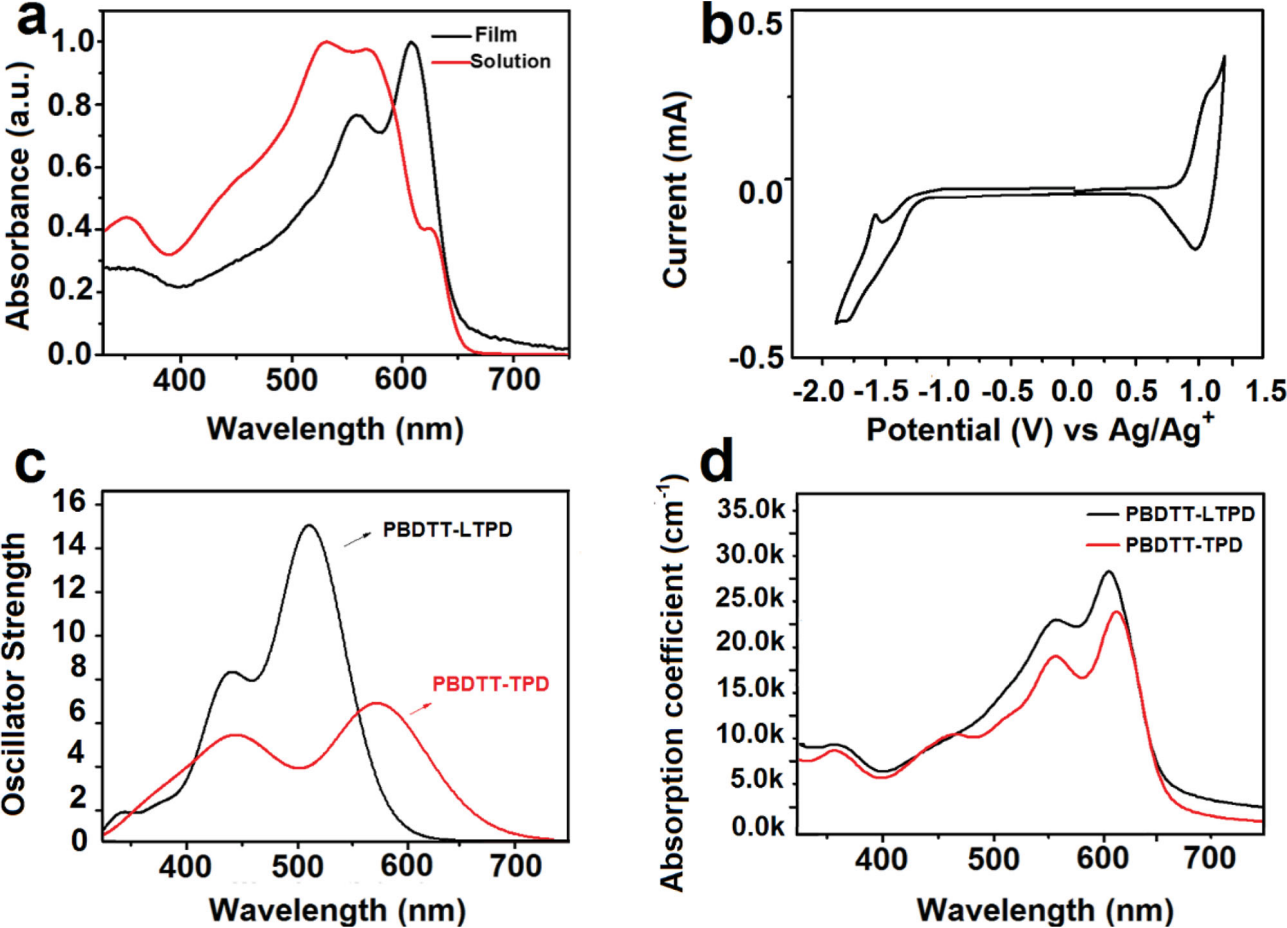
a) Absorption spectra of PBDTT‐LTPD in film and solution states. b) Cyclic voltammograms of polymer PBDTT‐LTPD films on a Pt electrode in 0.1 mol L^−1^ Bu_4_NPF_6_ (CH_3_CN) solution. c) Calculated optical spectra for the tetramers of PBDTT‐TPD and PBDTT‐LTPD and d) absorption spectra of PBDTT‐TPD and PBDTT‐LTPD film.

The experimental optical properties of PBDTT‐LTPD were characterized by UV–vis absorption spectroscopy, as presented in **Figure** **3**a. The shoulder at 625 nm of the solution should be attributed to the aggregation in solution state, thus demonstrating the strong interaction among main chains.^46^ This peak disappeared as the solution temperature rose to approximately 80 °C (Supporting information, Figure S2). The maximum absorption peak red‐shifted significantly from the solution to the film, and this occurrence should be ascribed to the π–π stacking in solid state. The calculations of optical properties of the tetramers for the two polymers were also carried out (Figure 3c). In the case of PBDTT‐TPD, two absorption peaks were found in the range of 350–700 nm. The first absorption peak was located at 558 nm, which comes from the first one excited state (S_1_). The second absorption peak was located at 451 nm, which comes from the S_5_ excited state. When going from PBDTT‐TPD to PBDTT‐LTPD, the absorption spectrum was blue‐shifted and two absorption peaks were also found. The lower‐energy peak was located at 514 nm and the higher‐energy peak was located at 436 nm, corresponding to S_1_ and S_5_ excited states, respectively (Figure 3c). The intensities of absorption peaks of tetramer of PBDTT‐LTPD were enhanced by 1.5–2 times compared with PBDTT‐TPD, indicating a better absorption ability of the PBDTT‐LTPD (see the details in Table S3, Supporting Information). This finding is consistent with the experimental results (Figure 3d), which may increase the *J*_sc_ values in corresponding PSC devices.

The electrochemical properties of the polymers were investigated using the CV method,^47^ as displayed in Figure 3b. The HOMO level of PBDTT‐LTPD was estimated to be −5.62 eV from the onset of oxidation. This HOMO level decreased because of the strengthened electron‐drawing capability of the linked‐acceptor monomer, which in turn was the result of the increase in acceptor density and of the backbone twisting caused by the substitute hexylthiophene bridge in the backbone. The LUMO level of PBDTT‐LTPD was estimated to be −3.43 eV from the onset of reduction potential. Thus, the electrochemical band gaps were calculated to be 2.19 eV (CV data was summarized in **Table** **1**). This linked‐acceptor polymer is expected to exhibit a high *V*_oc_ value in photovoltaic devices because of its low HOMO localization level.

**Table 1:** Molecular weight, optical, and electrochemical properties of the polymer

Polymer	*M_n_* [kDa]	PDI	UV–vis		CV		
			Solution *λ*_max_ [nm]	Film *λ*_max_ [nm]	*E*_HOMO_ [eV]	*E*_LUMO_ [eV]	*E_g_* [eV]
PBDTT‐LTPD	15.6	2.32	530	608	−5.62	−3.43	2.19

**Figure 4:**
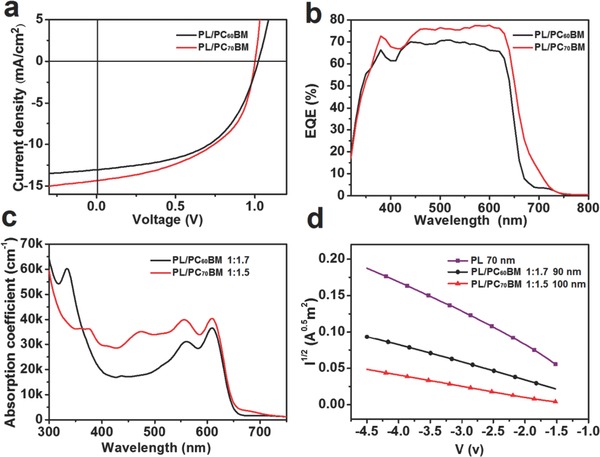
a) Ideal *J–V* characteristics of the PBDTT‐LTPD/PCBM‐based device. b) Corresponding EQE of the PBDTT‐LTPD/PCBM‐based device. c) Absorption coefficient of the PBDTT‐LTPD with PC_60_BM and PC_70_BM in film states and d) dark current densities for hole‐only devices composed of PBDTT‐LTPD, PBDTT‐LTPD:PC_60_BM (1:1.7 with 1% DIO), and PBDTT‐LTPD:PC_70_BM (1:1.5 with 1.5% DIO).

PSCs were fabricated with conventional and inverted BHJ structure by employing PBDTT‐LTPD as a donor with chloroform solution. To obtain detailed and convincing results, the devices based two different acceptor materials, namely, PC_60_BM and PC_70_BM, were compared with each other in conventional devices. The conventional device structure is indium tin oxide/poly(3,4‐ethylenedioxythiophene):poly(styrenesulfonate) (ITO/PEDOT:PSS, 40 nm)/polymer:PCBM/Ca(20 nm)/Al(100 nm) and the inverted device structure is ITO/ZnO(40 nm)/polymer:PCBM/MoO*_x_*(5 nm)/Ag(100 nm) with active areas measuring as 0.04 mm^2^. During device optimization, the influence of the ratios of the PBDTT‐LTPD to PCBM (w/w) and ratios of additive 1,8‐diiodooctane (DIO) in solutions (v/v) was studied. The performance of PBDTT‐LTPD conventional devices prepared under a typical condition is presented in **Table** **2**. The *V*_oc_ values of all of the devices can exceed 1 V. The optimized device that uses PC_70_BM as an acceptor can obtain a PCE of 7.59%, with a *V*_oc_ of 1.00 V, *J*_sc_ of 14.32 mA cm^−2^, and a FF of 52.0%. The PCE of the best inverted device was 7.45% under the same condition (see Table S2, Supporting Information). The devices that employ PC_60_BM as an acceptor can also attain a high PCE of 7.27% and a high *V*_oc_ of 1.02 V. **Figure** **4**a,b shows the best current density–voltage (*J–V*) curves and the external quantum efficiencies (EQE) of conventional devices (PBDTT‐LTPD is abbreviated as PL in these figures). Moreover, the other conventional device parameters for varied conditions are detailed in Table S1, Supporting Information.

**Table 2:** PSCs performances of the PBDTT‐LTPD conventional devices with 9 mg mL^−1^ CF solution

Acceptor	D:A [w/w]	DIO [v/v]	*J*_sc_ [mA cm^−2^]	*V*_oc_ [V]	FF [%]	PCE (max/avg.)a) [%]	Thickness [nm]
PC_60_BM	1:1.7	0%	8.93	1.02	57.8	5.47/5.44	100 (±5)
PC_60_BM	1:1.7	0.6%	11.96	1.02	53.7	6.63/6.48	100 (±3)
PC_60_BM	1:1.7	1.0%	13.01	1.02	53.6	7.27/7.17	95 (±2)
PC_70_BM	1:1.5	0%	4.74	1.01	46.9	2.34/2.28	120 (±5)
PC_70_BM	1:1.5	1.0%	13.44	1.01	50.4	7.08/6.96	110 (±3)
PC_70_BM	1:1.5	1.5%	14.32	1.00	52.0	7.59/7.43	120 (±3)
PC_70_BM	1:1.5	2.0%	13.61	1.01	50.0	7.06/7.02	110 (±5)

^a)^All the average PCE values are obtained from 8–12 cells of each condition.

The high *V*_oc_ values relative to those of PBDTT‐LTPD should be attributed to the low HOMO level caused by the linked‐acceptors that increased acceptor density and twisted the backbone. The increased *J*_sc_ values can be explained by the enhancement of the absorption coefficient. A broad coverage of 300–700 nm can be observed clearly in the EQE results (Figure 4b). The *J*_sc_ values calculated from the EQE curve are approximately 13.44 and 12.32 mA cm^−2^ for polymer/PC_70_BM‐ and polymer/PC_60_BM‐based devices, respectively. Figure 4c depicts the absorption coefficient of polymer/PC_60_BM and polymer/PC_70_BM blend films. The polymer/PC_70_BM exhibited significantly higher absorption capability than the polymer/PC_60_BM blend did. Thus, the polymer/PC_70_BM‐based devices should display high *J*_sc_ values.

Hole mobility was measured using the SCLC method given the device structure of ITO/PEDOT:PSS/polymer:PCBM/Au.^48^, ^49^ As illustrated in Figure 4d, the hole mobilities are 5.4 × 10^−5^, 3.55 × 10^−5^, and 1.95 × 10^−5^ cm^2^ V^−1^ s^−1^ for PBDTT‐LTPD, PBDTT‐LTPD:PC_60_BM, and PBDTT‐LTPD:PC_70_BM, respectively. The mobility of the polymer/PC_60_BM blend film was higher than that of the polymer/PC_70_BM blend film, which may explain why the FF values of polymer/PC_60_BM based PSCs were higher than those of polymer/PC_70_BM‐based PSCs.

**Figure 5:**
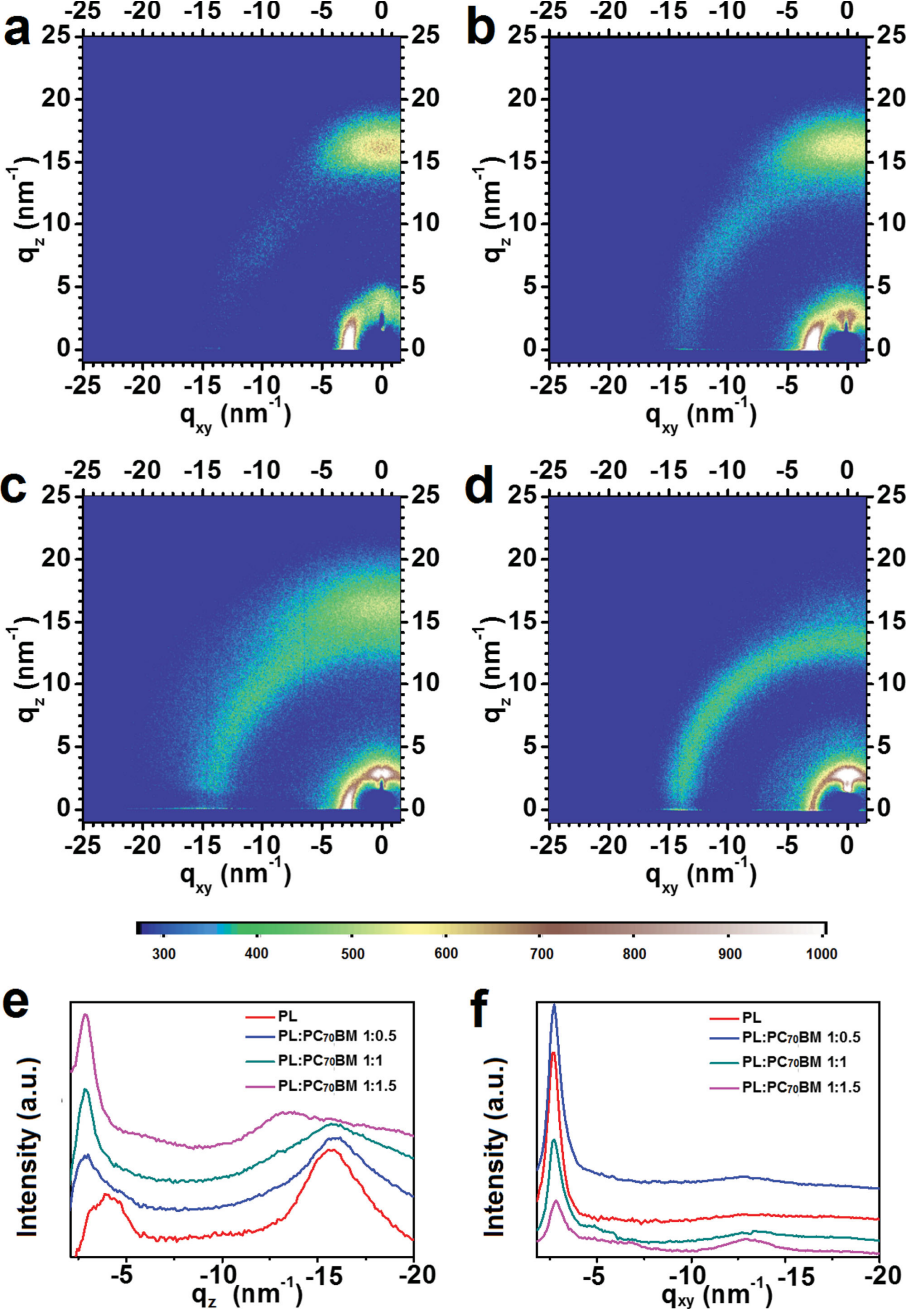
2D GIWAXS images. a) Neat PBDTT‐LTPD film. b) PBDTT‐LTPD:PC_70_BM (1:0.5) blend film. c) PBDTT‐LTPD:PC_70_BM (1:1) blend film. d) PBDTT‐LTPD:PC_70_BM (1:1.5) blend film. e) Out‐of‐plane patterns and f) in‐plane patterns for the 2D GIWAXS of films.

GIWAXS is used to determine the structural information and crystallinity in both neat and blend films (**Figure** **5**). Figure 5e,f shows the out‐of‐plane and in‐plane patterns of 2D GIWAXS profiles for neat PBDTT‐LTPD and PBDTT‐LTPD/PC_70_BM blend films, respectively. The π*–*π stacking peaks (010) are pronounced in the out‐of‐plane direction, which indicates a face‐on structure in the neat film. This structure is considered beneficial to hole transport.^50^, ^51^ The π*–*π stacking peaks gradually weaken when the amount of PC_70_BM incorporated into the blend increases. Thus, the hole mobility in polymer/PC_70_BM blend films may have decreased in comparison with that of neat films. This finding is consistent with the SCLC result. By contrast, the neat PBDTT‐LTPD film displayed a (100) peak at ≈3.95 nm^−1^, which is larger than that of the blend film. This result demonstrated that the exact blending of PCBM into polymer influenced molecular packing style and loosened the arrangement in the film. In another hand, the large π*–*π stacking distance (about 4.02 Å) maybe the main reason for the low fill factor. Figure 5a–d illustrates the change in stacking structure from face‐on one to a mixed face‐on one and edge‐on one, and to a predominately edge‐on one, as the amount of PC_70_BM increased. Although the PBDTT‐LTPD/PC_70_BM (1:1.5) film mainly possessed an edge‐on structure, it exhibited the best PSC performance. This finding should be attributed to the subtle balance between the film morphology and the appropriate D/A ratio.

**Figure 6:**
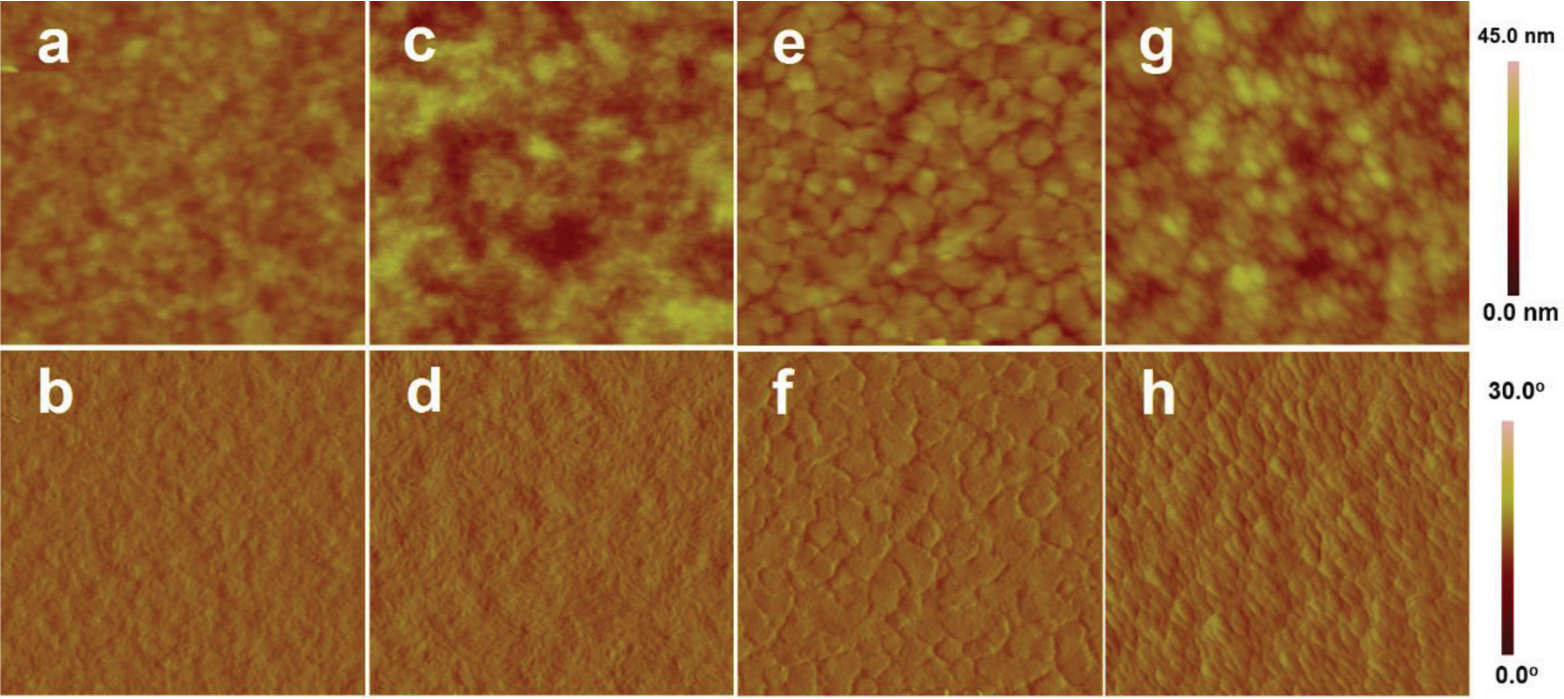
AFM topography images obtained in tapping mode: a) PBDTT‐LTPD:PC_60_BM (1:1.7) blend film, RMS: 1.20 nm. c) PBDTT‐LTPD:PC_60_BM (1:1.7 with 1% DIO) blend film, RMS: 1.70 nm. e) PBDTT‐LTPD:PC_70_BM (1:1.5) blend film, RMS: 1.80 nm. g) PBDTT‐LTPD:PC_70_BM (1:1.5 with 1.5% DIO) blend film, RMS: 2.60 nm. AFM phase images: b) PBDTT‐LTPD:PC_60_BM (1:1.7) blend film. d) PBDTT‐LTPD:PC_60_BM (1:1.7 with 1% DIO) blend film. f) PBDTT‐LTPD:PC_70_BM (1:1.5) blend film. h) PBDTT‐LTPD:PC_70_BM (1:1.5 with 1.5% DIO) blend film.

To examine the morphologies of the blend films further at different additive concentrations, AFM images were captured and depicted in **Figure** **6**. An improved and well‐defined nanostructure in the blend film is expected to enlarge the interfacial area for charge separation.^52^, ^53^, ^54^ The use of additives induces the formation of enhanced bicontinuous nanostructures in PBDTT‐LTPD:PCBM films. The PBDTT‐LTPD:PC_60_BM film with DIO (Figure 6d) exhibited significantly clearer fibrillar structures than the film lacking DIO did (Figure 6b) with the root mean square (RMS) roughness of 1.70 and 1.20 nm, respectively. Moreover, the domain of the PBDTT‐LTPD:PC_70_BM film with DIO (Figure 6h) was smaller than that of the film without DIO (Figure 6f) but with RMS roughness of 2.60 and 1.80 nm, respectively, which is beneficial to their PSC performance.

## 4 Conclusion

In summary, a novel conjugated polymer PBDTT‐LTPD composed of a linked‐acceptor LTPD unit was characterized and subject to facile synthesis. The introduction of the linked‐acceptor increases the electron‐withdrawing capability of the acceptor monomer and backbone twisting. As a result, the HOMO level decreases. The linked‐acceptor also enhanced the absorption coefficient of the polymer more than the single acceptor did. Thus, *V*_oc_ values exceeded 1 V were realized. Moreover, this linked‐acceptor polymer reported higher *J*_sc_ and PCE values than the single‐acceptor polymer did. This strategy efficiently increases the *V*_oc_ value of PSCs and may be used to design the donor materials for high‐performance PSCs in the future.

## 5 Experimental Section

^1^H NMR (400 MHz) and ^13^C NMR (400 MHz) spectra were obtained using a Bruker DMX‐400 NMR spectrometer with tetramethylsilane as an internal standard. MS spectra (MALDI‐TOF‐MS) were determined by a Micromass GCT‐MS spectrometer. UV–vis spectra were identified with a JASCO‐V570 spectrophotometer. Electrochemical CV was conducted on an electrochemical workstation (VMP3 Biologic, France) with a Pt disk coated with a molecular film, a Pt plate, and an Ag^+^/Ag electrode acting as the working, counter, and reference electrodes, respectively, in a 0.1 mol L^−1^ tetrabutylammonium phosphorus hexafluoride (Bu_4_NPF_6_) acetonitrile solution. The AFM images of the neat and blend films were captured on a Nanoscope Ia AFM (Digital Instruments) in tapping mode. The GIWAXS samples were prepared on PEDOT:PSS coated on ITO substrates following the same preparation conditions as those for devices. The data were obtained with an area pilatus 100k detector that had a resolution of 195 × 487 pixels (0.172 × 0.172 mm) at an in‐house X‐ray scattering facility (Xenocs WAXS/SAXS system). The X‐ray wavelength was 1.54 Å, and the incidence angle was 0.2°. The thickness of the active layer was measured by a Kla‐Tencor D120 profilometer. The *J–V* curves were determined by a Keithley 2420 source‐measure unit. The photocurrent was measured under illumination with an Oriel Newport 150 W solar simulator (AM 1.5 G), and the light intensity was calibrated with a Newport reference detector (Oriel PN 91150V). The EQEs of the devices were measured in air with an Oriel Newport system (Model 66902). The mobilities of the pristine and blend films were determined using a hole‐only SCLC method with the following diode structures for holes ITO/PEDOT:PSS/active layer/Au. *J–V* curves were assumed to be in the range of 0–5 V and the results were fitted to a space‐charge‐limited form.

The ground‐state geometries of the oligomers with four repeat units were optimized according to density functional theory (DFT) at the B3LYP/6–31G (d, p) level. For calculation, the alkyl groups were replaced by hydrogen and methyl groups. All of the calculations were performed in the gas phase with the Gaussian 09 program. Given that the combination of electron‐rich and electron‐deficient moieties in these oligomers is expected to generate a charge‐transfer characteristic in the low‐lying excitations, the low‐lying, optical excited states were evaluated by time‐dependent DFT with the long‐range corrected functional *ω*B97*x* and 6–31G (d, p) basis set.^55^ The *ω* values for this functional were optimized according to the method reported by Stein et al.^56^ The optimized *ω* values were equal to 0.09 and 0.11 bohr^−1^ for the PBDTT‐TPD and PBDTT‐LTPD tetrameters, respectively. Optical absorption spectra were simulated through a Gaussian broadening of the vertical transition energies and the associated oscillator strengths. The full width at half maximum was set to 0.1 eV.

PSC conventional devices with the structure of (ITO)/(PEDOT:PSS)/polymer:PCBM/Ca/Al were fabricated as follows: A 40 nm layer of PEDOT:PSS was spin‐coated onto a cleaned ITO‐coated glass substrate. The PBDTT‐LTPD/PCBM blend solution used for spin‐coating in this study was a 9 mg mL^−1^ chloroform solution. The PBDTT‐TPD/PCBM blend solution used for spin‐coating in this study was a 10 mg mL^−1^ ortho‐dichlorobenzene solution. The additive DIO was added prior to the spin‐coating process. PSC inverted devices with the structure of ITO/ZnO/polymer:PCBM/MoO*_x_*/Ag were fabricated as follows: The ZnO precursor solution was spin‐coated on top of the cleaned ITO‐coated glass substrate and the ZnO film thickness was approximately 30 nm. Then ZnO‐coated substrates were transferred into a glove box. The PBDTT‐LTPD/PC_71_BM blend solution used in this study for spin‐coating was 9 mg mL^−1^ chloroform solutions. The additive, 1,8‐octanedithiol (DIO), was added prior to spin‐coating process. The thickness of the active layer was controlled by altering the spin speed during this process. The devices were finished by evaporating metal electrodes with an area of 4 mm^2^. These areas were defined by masks. Furthermore, the layers were thermally evaporated at a pressure of 2 × 10^−6^ Torr. To optimize device performance, different acceptors (PC_60_BM or PC_70_BM), D/A weight ratios, and additive ratios (v/v) were applied during device fabrication.

## Supporting information

As a service to our authors and readers, this journal provides supporting information supplied by the authors. Such materials are peer reviewed and may be re‐organized for online delivery, but are not copy‐edited or typeset. Technical support issues arising from supporting information (other than missing files) should be addressed to the authors.

Supplementary
