# Supplementary material for: Linked‐Acceptor Type Conjugated Polymer for High Performance Organic Photovoltaics with an Open‐Circuit Voltage Exceeding 1 V
Source: Adv Sci (Weinh). 2015 Mar 13;2(4):1500021. doi: 10.1002/advs.201500021 (PMC5115348; doi:10.1002/advs.201500021)
Supplement: Supplementary file 1 — Supplementary [file ADVS-2-0l-s001.pdf]

Copyright WILEY-VCH Verlag GmbH & Co. KGaA, 69469 Weinheim, Germany, 2015.

Supporting Information

for *Adv. Sci.*, DOI: 10.1002/advs.201500021

Linked-Acceptor Type Conjugated Polymer for High Performance Organic Photovoltaics with an Open-Circuit Voltage Exceeding 1 V

Benzheng Xia, Kun Lu, Yifan Zhao, Jianqi Zhang, Liu Yuan, Lingyun Zhu, Yuanping Yi, and Zhixiang Wei**

Supporting information

Linked-acceptor Type Conjugated Polymer for High Performance Organic Photovoltaics with an Open-circuit Voltage Exceeding 1.0 V

Benzheng Xia, Kun Lu, Yifan Zhao, Jianqi Zhang, Liu Yuan, Lingyun Zhu, Yuanping Yi, and Zhixiang Wei**

Materials and synthesis. All reagents and chemicals were purchased from Aldrich, Alfa and used as received. Solvents and other common reagents were obtained from the Beijing Chemical Plant. Toluene, chloroform and tetrahydrofuran were freshly distilled prior to use. The other materials were used without further purification. 3, 4-dihexylthiophene (1), thiophene-3,4-dicarboxylic acid (4) was purchased from Aldrich and used as received. 5-octyl-thieno[3,4-*c*]pyrrole-4,6-dione (5) and 2,6-Bis(trimethyltin)-4,8-bis(5-(2-ethylhexyl) thiophen-2-yl)benzo[1,2-*b*:4,5-*b*]dithiophene (BDTT-Sn) were prepared according to the literature procedures.[1]

Compound (2). 3,4-dihexylthiophene (1) (2.0 g, 8.0 mmol) was dissolved in a mixture of chloroform and acetic acid (90 mL) (ratio 10:1). N-bromosuccinimide (NBS) (3.2 g, 18 mmol) was added to the solution in one portion. The reaction was stirred at ambient temperature for 3 h. The reaction solution was poured into water and extracted three times with chloroform. The organic phases were combined and the solvent was removed under reduced pressure. The crude product was purified by column chromatography using hexane as the eluent to afford the product as a colorless liquid (3.1 g, Yield: 95%). MS (MALDI-TOF-MS): m/z = 410.2. ^{1}H NMR (400 MHz, $CDCl_3$, ppm) δ: 2.53 (t, 4H), 1.65 (t, 4H), 1.38 (m, 12H), 0.92 (t, 6H).

Compound (3). 3,4-dihexyl-2,5-dibromothiophene (2) (1 g, 2.4 mmol) was poured into a 100 mL argon purged flask with 30 mL THF at –78 ^{o}C, and then *n*-butyllithium (2.4 M, 2.2 mL, 5.3 mmol) was added within 30 min. After the reaction was stirred for 3h at –78°C,

chlorotrimethylstannane (1.0 M in THF, 6 mL, 6 mmol) was added and the mixture was stirred for an additional 1 h at –78 ^{o}C. Then the mixture was stirred over night at ambient temperature. The mixture was poured into water and extracted three times with hexane. The organic phases were combined and the solvent was removed under reduced pressure to obtain the compound as yellow solid. The crude product was used for the next step without any purification.

Compound (6). 5-octylthieno[3,4-*c*] pyrrole-4,6-dione (5) (5 g, 18.8 mmol) was dissolved in a mixture of sulfuric acid (5 mL) and trifluoroacetic acid (50 mL). NBS (1.7 g, 9 mmol) was added in one portion to the solution and the reaction mixture was stirred at room temperature for 10 min. Then the brown-red solution was diluted with water (100 mL). The mixture was extracted three times with dichloromethane. The organic phases were combined and the solvent was removed under reduced pressure. The crude product was purified by column chromatography using dichloromethane/hexanes (1:1 ratio) as the eluent to finally get the product as white solid (2.6 g, Yield: 40%). MS (MALDI-TOF-MS): m/z = 344.2. ^{1}H NMR (400 MHz, $CDCl_3$, ppm) δ: 7.8 (s, 1H), 3.61 (t, 2H), 1.64 (m, 2H) 1.26 (m, 10H), 0.87 (t, 3H).

Compound (7). 3,4-dihexyl-2,5-bis-trimethylstannanyl-thiophene (3) (660.0 mg, 1.5 mmol) and 1-bromo-5-octyl-thieno[3,4-*c*]pyrrole-4,6-dione (6) (1.03 g, 3.0 mmol) and dry toluene (100 mL) were added to a 250 mL double-neck round-bottom flask. The solution was flushed with argon for 10 min. The flask was purged three times with successive vacuum and argon filling cycles and then $Pd(PPh_3)_4$ (30 mg) was added. The reaction was stirred over night at 95°C under argon. The reactant was cooled down to room temperature and the solvent was removed under reduced pressure. The crude product was purified by column chromatography using dichloromethane/hexanes (5:1 ratio) to get the product as yellow solid (934.0 mg, Yield: 80%). MS (MALDI-TOF-MS): m/z = 777.1. ^{1}H NMR(400 MHz, $CDCl_3$, ppm) δ:7.79 (s, 2H), 3.61 (t, 4H), 2.73 (t, 4H), 1.64 (m, 4H), 1.46 (m, 4H), 1.30 (m, 32H), 0.87 (t, 12H).

Compound (8). 2,5-bis(5-octyl-thieno[3,4-*c*]pyrrole-4,6-dione-1-yl)3,4-dihexylthiophene (7) (500 mg, 0.64 mmol) was dissolved in trifluoroacetic acid (30 mL). NBS (249.6 mg, 1.41 mmol) was added in one portion to the solution and the reaction mixture was stirred at room temperature for 4 h. Then the brown-red solution was diluted with water (100 mL). The mixture was extracted with dichloromethane. The organic phases were combined and the solvent was removed under reduced pressure. The crude product was purified by column chromatography using dichloromethane/hexanes (3:1 ratio) to get the product as yellow solid (589.9 mg, Yield: 98%). MS (MALDI-TOF-MS): m/z = 936.9. ^{1}H NMR (400 MHz, $CDCl_3$, ppm) δ: 3.61 (t, 4H), 2.73 (t, 4H), 1.64 (m, 4H), 1.46 (m, 4H), 1.30 (m, 32H), 0.87 (t, 12H). ^{13}C NMR (101 MHz, $CDCl_3$) δ: 162.28, 161.99, 145.56, 139.14, 135.61, 132.23, 128.57, 113.41, 39.66, 32.69, 32.27, 31.45, 30.31, 30.04, 30.02, 29.24, 29.18, 27.78, 23.52, 23.46, 14.97, 14.93.

Synthesis of PBDTT-LTPD. 2,5-bis(3-bromo-5-octyl-thieno[3,4-*c*]pyrrole-4,6-dione)3,4-dihexylthiophene (8) (93.7 mg, 0.1 mmol) and 2,6-bis(trimethyltin)-4,8-bis(5-(2-ethylhexyl)thiophen-2-yl)benzo[1,2-*b*:4,5-*b*]dithiophene (BDTT-Sn) (90.4 mg, 0.1 mmol) were dissolved in toluene (10 mL) and DMF (2mL). The solution was flushed with argon for 10 min, and then Pd $(PPh_3)_4$ (11.5 mg, 0.01 mmol, 10% with respect to the monomer) and the flask was purged three times with successive vacuum and argon filling cycles. The polymerization reaction was heated to 100°C, and the mixture was stirred for 10 h under argon atmosphere. The mixture was cooled to room temperature and poured slowly in methanol (200 mL). The solid was filtered through 0.45 μm PTFE filter. Finally the polymer was extracted with chloroform. The polymer was further purified by column chromatography using chloroform as the eluent. Then the polymer solution was poured in methanol (200 mL). The solid was filtered through 0.45 μm PTFE filter and a dark red solid was obtained. The solid was dried under vacuum overnight (87.8 mg, Yield: 65%), M_n=112.1 kDa, PDI = 1.67,

^{1}H NMR (400 MHz, $CDCl_3$, ppm) δ: 7.35-7.74 (r, 4H), 6.90-6.94 (r, 2H), 3.53-3.61 (r, 4H), 2.73-2.92 (r, 8H), 1.30-1.78(r, 80H), 0.86-0.88 (r, 18H).

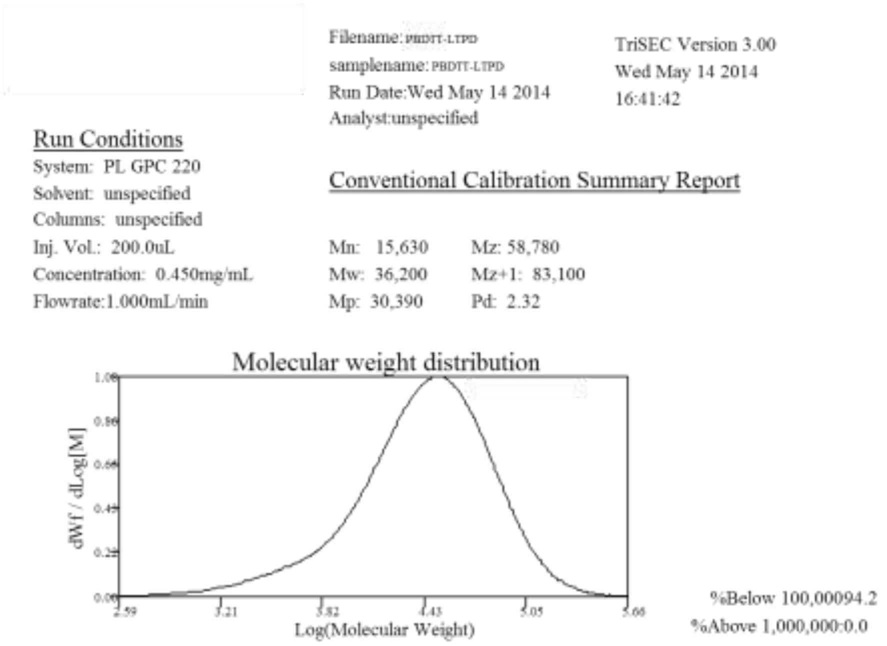

Figure S1. The high-temperature GPC measurement using 1,2,4-trichlorobenzene as the eluent (140 °C) for PBDTT-LTPD.

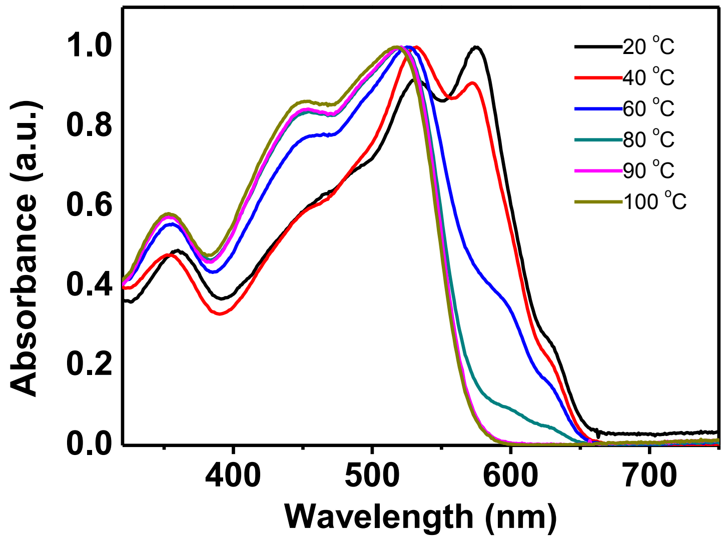

Figure S2. UV absorption spectra of PBDTT-LTPD in 1,2-Dichlorobenzene during heating process from 20 to 100 °C

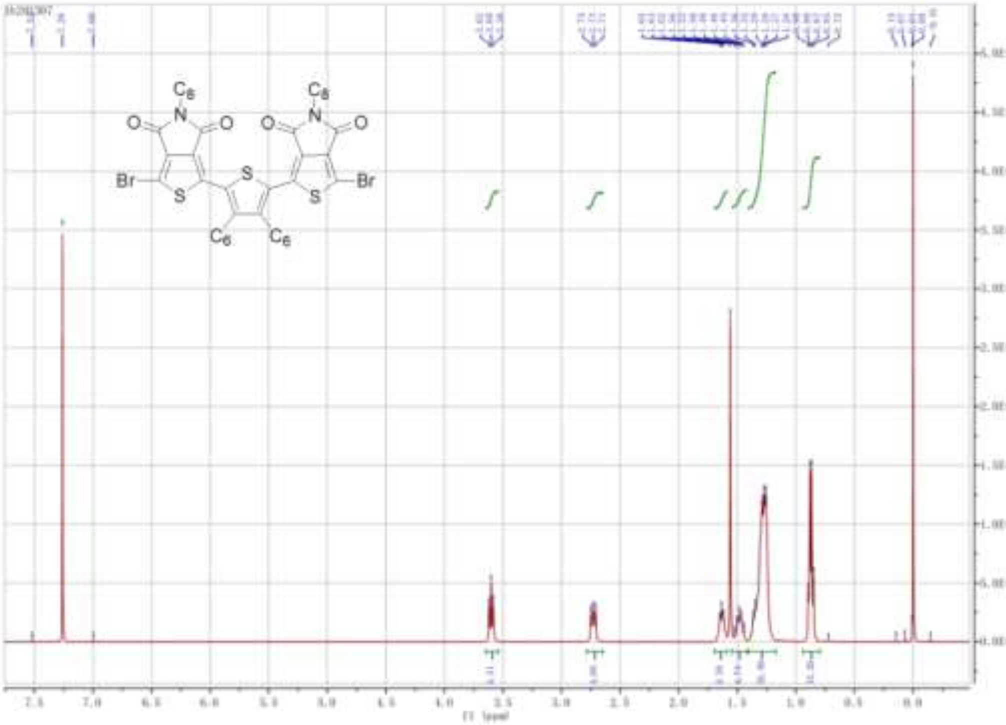

Figure S3. ^{1}H NMR spectra of monomer (Compound 8).

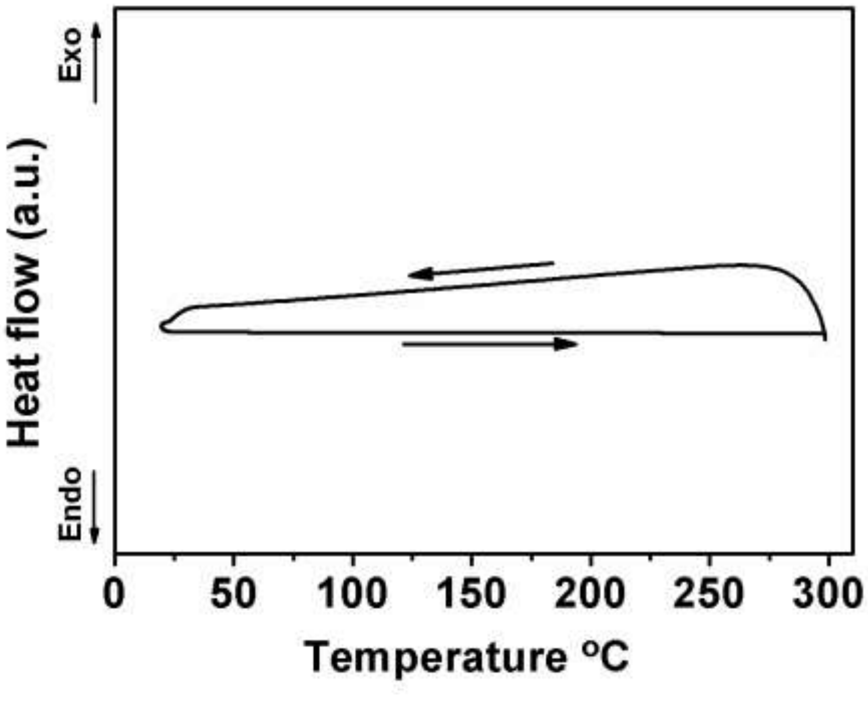

Figure S4. DSC curve of PBDTT-LTPD.

Table S1. PSC performances of the PBDTT-LTPD devices spin-coated with 9 mg/mL CF solution.

Acceptor	D:A [w/w]	DIO [v/v]	J_{sc} [mA cm^{-2}]	V_{oc} [V]	FF [%]	PCE [%]
$PC_{60}BM$	1:1.4	0.8 %	9.72	1.02	59.2	5.91
$PC_{60}BM$	1:1.5	0.8 %	11.32	1.02	55.1	6.49
$PC_{60}BM$	1:1.7	0.8 %	11.23	1.02	55.7	6.51
$PC_{60}BM$	1:1.9	0.8 %	12.08	1.01	54.7	6.75
$PC_{60}BM$	1:1.7	0.6 %	11.96	1.02	53.7	6.63
$PB_{60}BM$	1:1.7	1.0 %	13.01	1.02	53.6	7.27
$PC_{70}BM$	1:1.1	1.0%	11.47	1.01	49.3	5.89
$PC_{70}BM$	1:1.2	1.0%	13.41	1.00	48.6	6.71
$PC_{70}BM$	1:1.5	1.0%	13.44	1.01	50.4	7.08
$PC_{70}BM$	1:1.8	1.0%	12.62	1.00	51.7	6.70
$PC_{70}BM$	1:2	1.0%	12.57	0.99	49.9	6.41
$PC_{70}BM$	1:2.5	1.0%	11.85	0.99	50.6	6.12
$PC_{70}BM$	1:3	1.0%	11.94	1.00	48.6	5.98
$PC_{70}BM$	1:1.5	1.5%	14.32	1.00	52.0	7.59
$PC_{70}BM$	1:1.5	2.0%	13.61	1.01	50.0	7.06
$PC_{70}BM$	1:1.5	2.5%	13.43	1.00	49.7	6.91

Table S2. The performance of inverted type polymer solar cells of the PBDTT-LTPD.

Acceptor	D:A [w/w]	DIO [v/v]	J_{sc} [mA cm^{-2}]	V_{oc} [V]	FF [%]	PCE (max/avg.)* [%]	Thickness [nm]
$PC_{70}BM$	1:1.5	1.0%	12.64	0.79	41.6	4.32 / 4.13	110 (±5)
$PC_{70}BM$	1:1.5	1.2%	13.40	0.90	46.3	5.86 / 5.70	120 (±3)
$PC_{70}BM$	1:1.5	1.5%	13.51	0.96	54.7	7.45 / 7.26	120 (±2)
$PC_{70}BM$	1:1.5	1.8%	12.91	0.95	47.4	6.07 / 5.97	95 (±3)
$PC_{70}BM$	1:1.2	1.5%	12.03	0.95	42.4	5.09 / 4.71	110 (±5)
$PC_{70}BM$	1:1.7	1.5%	12.64	0.79	41.6	6.17 / 5.92	120 (±3)

*All the average PCE are obtained from 8–12 cells of each condition.

Table S3. TD/optimized-ωB97x/6-31G (d,p)-calculated excitation energies and oscillator strengths of the tetramers for PBDTT-TPD and PBDTT-LTPD.

	PEAK 1				PEAK 2			
Polymer	Excited state	Excitation Energy	Transition Dipole Moment	Oscillator Strength	Excited state	Excitation Energy	Transition Dipole Moment	Oscillator Strength
PBDTT-TPD	S_1	2.22 eV (558 nm)	4.64(Debye)	2.7670	S_5	2.75 eV (451 nm)	3.90 (Debye)	1.0260
PBDTT-LTPD	S_1	2.41 eV (514 nm)	24.43 (Debye)	5.4685	S_5	2.84 eV (436 nm)	18.11(Debye)	1.5033

[1]. L. Huo, S. Zhang, X. Guo, F. Xu, Y. Li and J. Hou, *Angew. Chem. Int. Ed.* **2011**, *123*, 9871.
